# Kidney outcomes associated with SGLT2 inhibitors compared to other glucose-lowering drugs: a real-world study from China

**DOI:** 10.3389/fphar.2024.1468435

**Published:** 2024-12-03

**Authors:** Xiang Xiao, Shuming Ji, Tao Zheng, Tianzhu Wang, Dapeng Jiang, Fang Liu

**Affiliations:** ^1^ Division of Nephrology, West China Hospital of Sichuan University, Chengdu, China; ^2^ Department of Nephrology, The first affiliated hospital of Chengdu Medical college, Chengdu, China; ^3^ Laboratory of Diabetic Kidney Disease, Centre of Diabetes and Metabolism Research, West China Hospital of Sichuan University, Chengdu, China; ^4^ Department of Project Design and Statistics, West China Hospital, Sichuan University, Chengdu, China; ^5^ Department of Clinical Research Management, West China Hospital, Sichuan University, Chengdu, China

**Keywords:** type 2 diabetes, diabetic kidney disease, sodium-glucose transport protein 2 (SGLT2) inhibitors, real world study, outcomes

## Abstract

**Objective:**

This study aimed to investigate the association between the utilization of Sodium-dependent glucose cotransporters inhibitors (SGLT2i) in real-world settings and kidney outcomes in patients with type 2 diabetes (T2D) and chronic kidney disease (CKD) in mainland China.

**Methods:**

In a retrospective analysis of electronic medical records from West China Hospital of Sichuan University, patients with T2D and CKD were included. Patients were divided into two groups, those initiating treatment with SGLT2i and those receiving other glucose-lowering drugs (oGLDs). The primary focus lies in examining the impact of SGLT2i on the decline slope of eGFR and major kidney events in these patients.

**Results:**

We enrolled 944 patients diagnosed with both T2D and CKD. Out of these, 605 patients were prescribed SGLT2i, while the remaining 339 patients received oGLDs. The median follow-up duration were 16.8 months and 20.6 months, respectively. Throughout the follow-up period, we observed a significant decrease in the rate of eGFR decline in patients using SGLT2i (4.94 mL/min/1.73 m^2^ per year reduction compared to oGLDs, 95% CI: 4.73–5.15). A total of 101 kidney composite endpoint events occurred, with 31 events in the SGLT2i group and 70 events in the oGLDs group. The use of SGLT2i was associated with a 65% decrease in the risk of kidney composite endpoint events (hazard ratio 0.35, 95% CI 0.19–0.63).

**Conclusions:**

In clinical practice, SGLT2i have shown favorable effects on kidney prognosis in patients with T2D and CKD in mainland China. These effects remain consistent across patients with varying risks of CKD progression.

**Clinical Trial Registration Number:**

ChiCTR2300068497.

## 1 Background

Approximately 40% of individuals with diabetes experience kidney impairment, making it a leading cause of chronic kidney disease (CKD) worldwide (Alicic et al., 2017). Recent data reveals that the prevalence of CKD among the Chinese population is 8.2%, with 31.3% of patients also having diabetes (Wang et al., 2023). Compared to the previous national survey, there has been a 2.6% increase in the proportion of CKD cases associated with diabetes (Wang et al., 2023). Globally, the prevalence of type 2 diabetes (T2D) and CKD is also steadily increasing, leading to substantial societal and economic burdens (Afkarian et al., 2016; Zhang et al., 2016; Zhang et al., 2020).

Sodium-dependent glucose cotransporters inhibitors (SGLT2i) have consistently demonstrated a clear renoprotective effect in multiple randomized controlled trials (RCTs), including reducing proteinuria, slowing estimated glomerular filtration rate (eGFR) decline, and lowering the risk of end-stage renal disease (ESRD) (Perkovic et al., 2019; Heerspink et al., 2020a; Herrington et al., 2023). Based on the available evidence from RCTs, SGLT2i have been recommended by guidelines for the treatment of patients with T2D and CKD (Navaneethan et al., 2023; Navaneethan et al., 2021; de Boer et al., 2022). These recommendations highlight the expanding indications for SGLT2i therapy, emphasizing its effectiveness, safety, and significance in improving kidney outcomes. However, due to the strict inclusion criteria of RCTs, the data collected can only represent a small portion of the ideal world and may differ significantly from the patients encountered by doctors in actual clinical settings. As a result, an increasing number of real-world studies (RWS) are being conducted to explore the response of SGLT2i in real clinical practice (Forbes et al., 2023).

The significance of supplementing RCTs findings with real-world data is increasingly recognized. This approach allows for the exploration of the effectiveness of medications in real-world settings and the application of clinical trial results to diverse patient populations, particularly those at lower or higher risk. In fact, an increasing number of RWS has revealed significant variations in patient response to SGLT2i within clinical practice (Nagasu et al., 2021; Forbes et al., 2023). Furthermore, variations in healthcare standards, patient health awareness, healthcare insurance coverage, and significant heterogeneity in patient response to different medications within different regions may contribute to discrepancies in findings regarding different RWS. Additionally, there is currently limited research investigating the actual treatment effectiveness of SGLT2i in mainland China among patients with T2D and CKD.

Therefore, this study aims to investigate the impact of SGLT2i on kidney outcomes in patients with T2D and CKD in mainland China, particularly considering varying risks of CKD progression. Additionally, this study seeks to explore potential differences in treatment outcomes among different subgroups of patients.

## 2 Methods

### 2.1 Study participants

A retrospective cohort study was conducted at Sichuan University Hospital from January 2019 to May 2022. The study included patients with both T2D and CKD who received medical care either as inpatients or outpatients. During this period, patients were prescribed either SGLT2i (Empagliflozin or Dapagliflozin) or a novel oGLD as an addition to their existing treatment. The index drug refers to the initial or added treatment, while the index time is the defined period. The cohort dataset consists of demographic information, medication usage, diagnostic data, and other variables sourced. Inclusion and exclusion criteria were detailed in the Supplementary. All medical data processing adhered to established standards and knowledge repositories, including the Electronic Medical Record Basic Dataset (2014), Health Level Seven (HL7) Clinical Document Architecture, Release 2, HL7 Reference Information Model Vers. 2.07, National Standard GB/T Terminology Set, International Classification of Diseases-10 (ICD-10), LOINC, and the Health Record Data Model.

This study obtained approval from the Ethics Committee of West China University of Sichuan (No: 20221520), and was duly registered on the Chinese Clinical Trial Registry (No: ChiCTR2300068497), in strict adherence to the principles outlined in the Declaration of Helsinki. In accordance with the legal requirements in China, a retrospective study was conducted, following the prescribed procedures for obtaining exemption from informed consent.

### 2.2 Study design

The on-treatment time refers to the time from the index date to 1) discontinuation of the index drug, 2) initiation of another new hypoglycemic medication or SGLT2i, 3) patient’s departure from the medical institution or database, or 4) the time of the last data collection (whichever occurs first). The intention-to-treat (ITT) time refers to the time from the index time to the patient’s departure from the medical institution or database, the last data collection date, or death (whichever occurs first). Similar to the methodology defined in previous studies (Nagasu et al., 2021; Heerspink et al., 2020b). The on-treatment time served as the primary time scale for analyzing eGFR slope changes. The ITT follow-up was used for time-to-major kidney events analysis.

### 2.3 Indicators measurements and endpoints defined

Serum creatinine was measured using an enzymatic method. The eGFRwas calculated using the Chronic Kidney Disease Epidemiology Collaboration (CKD-EPI) equation (Kidney Disease: Improving Global Outcomes KDIGO Glomerular Diseases Work Group, 2021). Urine protein was qualitatively detected using a strip test method and recorded as (-, ±, 1+, 2+, 3+, 4+). Proteinuria was defined as exceeding 1+, where 1+ reaction corresponds to a urine protein level greater than 30 mg/dL. Urine creatinine was detected using the creatinine oxidase method. Total urine protein was determined using the endpoint method. Hematuria in urine was defined as the presence of an average of three or more red blood cells in the field of view under high magnification. Rapid eGFR decline was defined as a yearly decrease of eGFR greater than 3 mL/min/1.73 m^2^. Due to the frequent absence of Albumin-to-Creatinine Ratio (ACR) measurements, which may introduce significant bias if imputed, we aimed to establish a correlation between the qualitative findings of urine protein in routine urinalysis and the severity of ACR. In routine urinalysis, a negative or ± result indicates the absence or mild albuminuria, while a 1+ or 2+ result signifies a moderate increase, and a 3+ or 4+ result signifies a severe increase in albuminuria. Subsequently, it was combined with eGFR levels to classify the patients' risk for CKD progression as low, moderate, high, or very high risk (KDIGO, 2012).

The primary outcome was defined as the change in eGFR slope after the use of SGLT2i or oGLDs. Secondary outcome measures were defined as follows: 1) eGFR <15 mL/min/1.73 m^2^ (ESRD), 2) eGFR decline exceeding 30% from baseline, 3) kidney composite endpoint defined as ESRD or eGFR decline exceeding 30% from baseline. In the analysis of the kidney composite endpoint, if a participant experiences more than one event, the first to occur was considered as the outcome.

### 2.4 Statistical analysis

Study population was characterized by using mean ± SD and proportions as appropriate, and the inverse probability of treatment weighting (IPTW) using variables that might have affected treatment assignment or outcomes was developed to predict the likelihood a T2D or CKD patient would be prescribed SGLT2i. Patients in the two treatment groups were weighted by IPTW, and the standardized mean difference (SMD) before weighted and after were described respectively. An imbalance was considered nonnegligible if a SMD of >10% was present between the two groups after IPTW. The eGFR trajectory from pre-index to post-index date was displayed graphically over time, and each monthly time point was represented with the eGFR value closest to the time point of interest, within a defined interval. Time zero was indicative of the estimated intercept of the pre-index slopes. In order to assess the differences between post-index date eGFR slopes for patients taking SGLT2i and slopes for those oGLDs, we developed a linear mixed regression model, where treatment groups (SGLT2i or oGLDs), time (linear), and the interaction between treatment group and time were included as fixed factors. Further to estimate the heterogeneity in the associations between the use of SGLT2i, eGFR slope by each subgroup categorized according to sex, age, rapid eGFR decline, hematuria, the use of RAASi, eGFR or risk of CKD progression with a statistically significant interaction was defined as *p*-value < 0.05. And we also conducted the subgroup comparison noted above. In the analysis of the kidney endpoint events, the Cox regression model was employed to examine the association between different drug usage and major kidneyl endpoint events. For the analysis of subgroup endpoint events, the IPTW method was once again utilized to balance the propensity scores of variables among patients in the groups, followed by Cox regression analysis. Multiple interpolation method to address missing data. A statistically significant interaction was defined as *p*-value < 0.05. All statistical analyses were performed with R version 4.3.1 software. Statistical significance was defined as a *p*-value < 0.05 using two-sided tests.

## 3 Result

### 3.1 Baseline characteristics

Prior to propensity score analysis, the study included 944 T2D and CKD patients. Among them, 605 patients received SGLT2i treatment, while 339 patients received novel oGLDs treatment (Supplementary Figure S1). Variables with SMD exceeding 10% included age, serum albumin, hemoglobin, total cholesterol, eGFR at index time, prescription of dipeptidyl peptidase-4 inhibitors (DPP4i), α-glucosidase inhibitors (AGI), sulfonylureas, glucagon-like peptide-1 receptor agonists (GLP-1RA), thiazolidinediones, and insulin, as well as the distribution of patients across different eGFR levels at index time. Compared to oGLDs patients, SGLT2i patients were younger with higher levels of serum albumin, hemoglobin, and total cholesterol. They had lower proportions of DPP-4i, sulfonylurea, AGI, GLP-1RA and insulin usage. Average eGFR and proportion of patients with eGFR ≥60 mL/min/1.73 m^2^ were higher, while proportion of patients with eGFR <45 mL/min/1.73 m^2^ was lower in SGLT2i group compared to oGLDs group (Supplementary Table S1).

In the PS-adjusted analysis using IPTW, the following variables were considered: age, sex, HbA1c, serum albumin, hemoglobin, total cholesterol, triglycerides, LDL-cholesterol (LDL-c), eGFR, eGFR stratification, hypertension, hematuria, proteinuria classification, insulin, oral hypoglycemic drugs, Angiotension Converting Enzyme inhibitors (ACEi), Angiotensin Receptor Blocker (ARB), statins, and the pre-treatment eGFR decline slope. After processing, the SMD for all baseline variable characteristics were <9.9% (Table 1). The average age was 62 years, with males accounting for 65.8%. The mean HbA1c was 7.17%, and the average eGFR was 68.87 mL/min/1.73 m^2^. Patients with eGFR <60 mL/min/1.73 m^2^ accounted for 39.1%, while those with eGFR <45 mL/min/1.73 m^2^ accounted for 21.0%. The proportion of patients with hematuria was 64.8%. The average pre-treatment eGFR decline slope was 3.07 ± 13.50 mL/min/1.73 m^2^ per year, with ACEi usage at 6.0% and ARB usage at 33.9%. The proportion of patients with proteinuria - or ± was 51.7%, while the proportion of patients with proteinuria 1+ or 2+ was 36.9%. Additionally, the proportion of patients with proteinuria 3+ or 4+ was 11.4% (Supplementary Table S1).

**TABLE 1:** Clinical characteristics at index date after IPTW (Propensity Score).

Characteristics	SGLT2i group (n = 605)	Other glucose-lowering drugs group (n = 339)	SMD (%)
Age, mean (SD), years	62.45 (13.55)	62.14 (13.89)	2.3
Gender, Male, [n (%)]	318 (31.8)	276 (32.1)	0.8
Hemoglobin A1c, mean (SD), %	7.21 (1.32)	7.34 (1.55)	8.9
Serum albumin, mean (SD), g/L	43.66 (5.36)	44.18 (5.14)	9.9
Hemoglobin, mean (SD), g/L	131.72 (22.52)	132.53 (22.01)	3.6
Triglyceride, mean (SD), mmol/L	4.39 (1.24)	4.47 (1.58)	5.6
Total cholesterol, mean (SD), mmol/L	2.05 (2.11)	2.00 (1.58)	2.7
LDL-c, mean (SD), mmol/L	2.46 (0.99)	2.51 (1.22)	4.3
ACEi, [n (%)]	63 (6.3)	46 (5.3)	4.3
ARB, [n (%)]	348 (34.8)	286 (33.3)	3.2
Statin, [n (%)]	382 (38.2)	310 (36.0)	4.5
Proteinuria classification [n (%)]	\	\	7.8
− or ±	525 (52.6)	485 (56.4)	
1+ or 2+	385 (38.5)	301 (35.0)	
3+ or 4+	90 (9.0)	75 (8.7)	
Hematuria, [n (%)]	659 (65.9)	541 (62.8)	6.4
Glucose-lowering drugs, [n (%)]			
Metformin	467 (46.7)	422 (49.1)	4.7
DPP4i	381 (38.1)	360 (41.8)	7.6
Sulfonylurea	273 (27.3)	271 (31.5)	9.2
GLP-1RA	27 (2.7)	18 (2.1)	3.9
Thiazolidinedione	234 (2.3)	20 (2.3)	<0.1
AGI	188 (18.8)	134 (15.5)	8.8
Insulin	317 (31.7)	280 (32.6)	1.9
Hypertension, [n (%)]	443 (44.3)	364 (42.3)	4.0
eGFR, mL/min/1.73 m^2^	69.07 (26.33)	70.46 (31.55)	4.8
eGFR ≥60 mL/min/1.73 m^2^, [n (%)]	599 (60.0)	537 (62.4)	5.0
eGFR <60 mL/min/1.73 m^2^, [n (%)]	400 (40.0)	324 (37.6)	5.0
eGFR 45–59 mL/min/1.73 m^2^, [n (%)]	166 (16.6)	137 (15.9)	2.0
eGFR <45 mL/min/1.73 m^2^, [n (%)]	234 (23.4)	187 (21.7)	4.1
Rate of eGFR change prior to index, mean (SD), mL/min/1.73 m^2^ per year	−3.29 (13.84)	−2.62 (13.54)	4.9

IPTW, inverse probability of treatment weighting; SGLT2i, Sodium-glucose cotransporter-2, inhibitors; SMD, standardized mean difference; SD, standard deviation; ACEi, Angiotension Converting Enzyme inhibitors; ARB, angiotensin receptor blocker; eGFR, estimated Glomerular Filtration Rate; LDL-c, low density lipoprotein cholesterol; DPP4i, dipeptidyl peptidase-4, inhibitors; GLP-1RA, glucagon-like peptide-1, receptor agonists; AGI, α-glucosidase inhibitor; A standardized difference >10% is considered a nonnegligible difference.

### 3.2 Primary outcome

**FIGURE 1:**
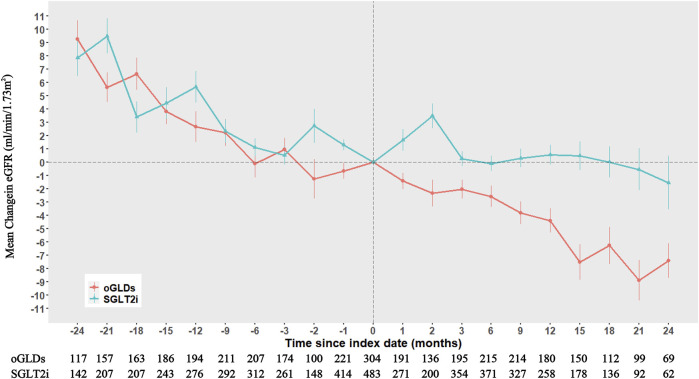
Temporal eGFR changes before and after the initiation of SGLT2i and oGLDs (On time treatment analysis).

**FIGURE 2:**
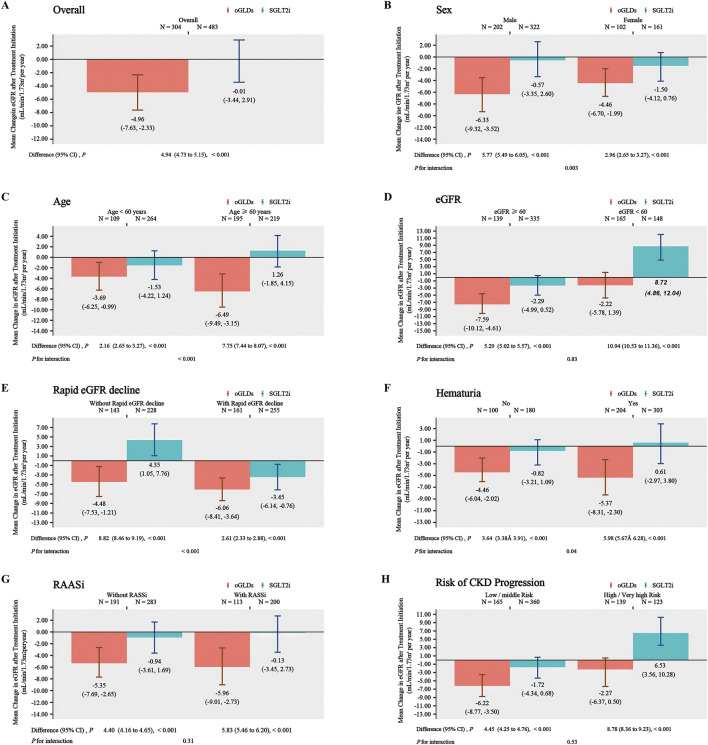
The annual eGFR slope in subgroups following the initiation of SGLT2i and oGLDs (On time treatment analysis): all **(A)**, sex (“Female” vs “Male”) **(B)**, age (“<60” vs “≥60” years) **(C)**, eGFR (“<60” vs “≥60” mL/min/1.73 m^2^) **(D)**, rapid eGFR decline (“yes” vs “no”) **(E)**, RBC (“<3” vs “≥3”/HP) **(F)**, RAASi (“yes” vs “no”) **(G)**, risk of CKD progression (“low or middle” vs “high or very high”) **(H)**.

The follow-up duration for patients in the SGLTi group and oGLDs group was 16.8 and 19.4 months, respectively. Prior to index time, the mean eGFR change rates were 3.29 ± 13.84 mL/min/1.73 m^2^ per year for the SGLT2i and 2.62 ± 13.54 mL/min/1.73 m^2^ per year for the oGLDs. The temporal changes in eGFR before and after administration of SGLT2i or oGLDs are showed in Figure 1. In the on-treatment time analysis, the mean eGFR decline slopes for the SGLT2i group and oGLDs group were 0.02 mL/min/1.73 m^2^ per year (95% CI, −2.9–3.4) and 4.96 mL/min/1.73 m^2^ per year (95% CI, 2.33–7.63), respectively. The between-group difference in eGFR decline rate was 4.94 mL/min/1.73 m^2^ per year (4.73–5.15), favoring the SGLT2i (*p* < 0.001) (Figure 2A). The temporal changes in eGFR before and after administration of SGLT2i or oGLDs, stratified by risk of CKD progression, are illustrated in Supplementary Figure S2. Among all subgroups, patients using SGLT2i exhibited a significantly slower decline in eGFR compared to those using oGLDs (all *p* < 0.05) (Figures 2B–H). No interaction was observed between SGLT2i and the use of RAASi (“yes” vs “no”) or eGFR (“<60” vs “ ≥60”mL/min/1.73 m^2^) or risk of CKD progression (“low or middle” vs “high or very high”) prior to treatment initiation (all *p* > 0.05) (Figures 2B–H). However, interactions were observed between SGLT2i and sex (“Female” vs “Male”), age (“<60” vs “≥60” years), rapid eGFR decline (“yes” vs “no”), and hematuria (“yes” vs “no”) (all *p* < 0.05) (Figures 2B–H). Similar findings were observed in the ITT population, as depicted in Supplementary Figures S3–S5.

### 3.3 Secondary outcome

**FIGURE 3:**
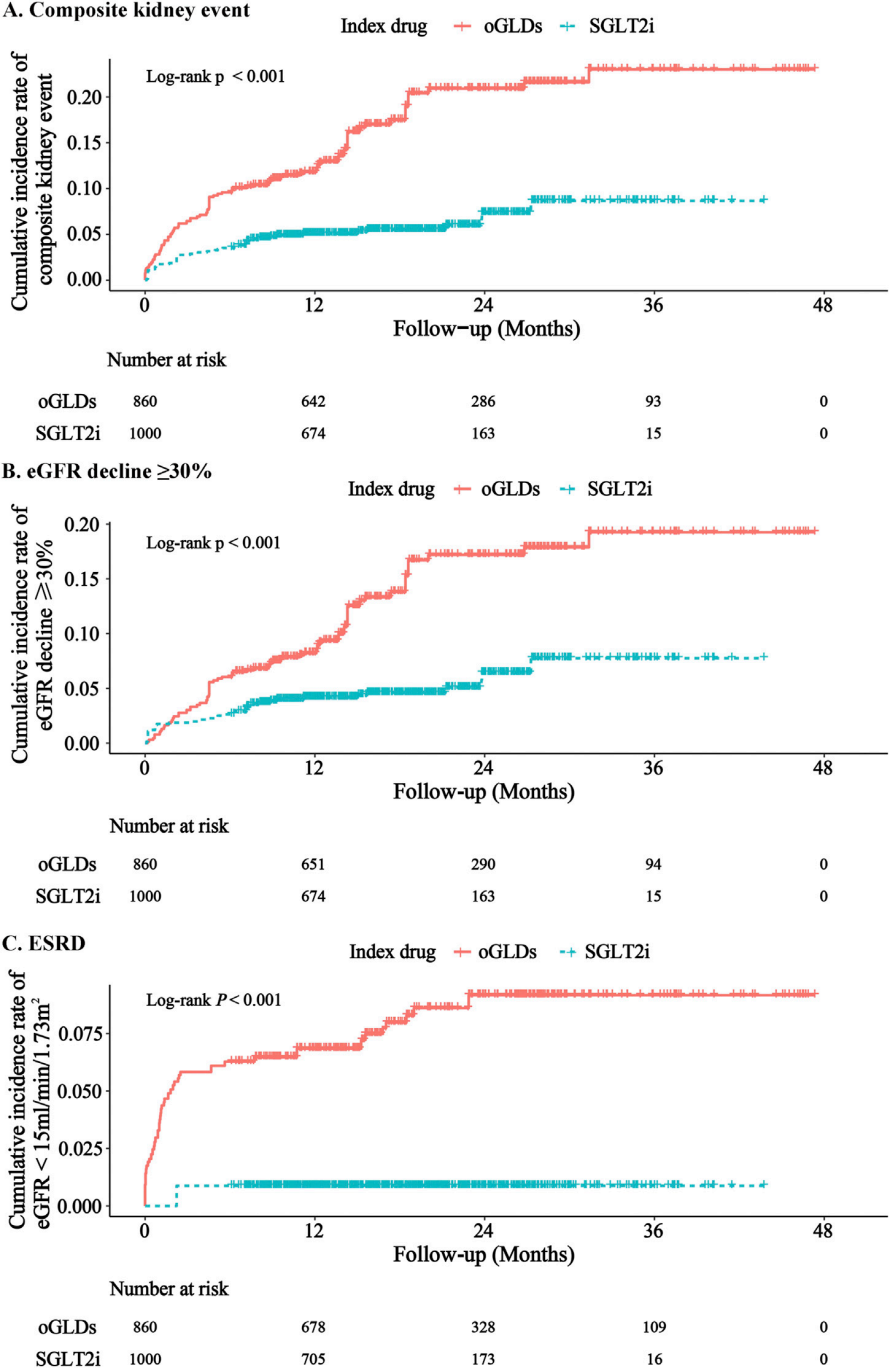
Cumulative incidence rates of major renal events following initiation of SGLT2i and oGLDs (ITT analysis): The cumulative incidence rates of kidney composite events **(A)**, eGFR decline ≥30% **(B)**, and ESRD **(C)**.

**FIGURE 4:**
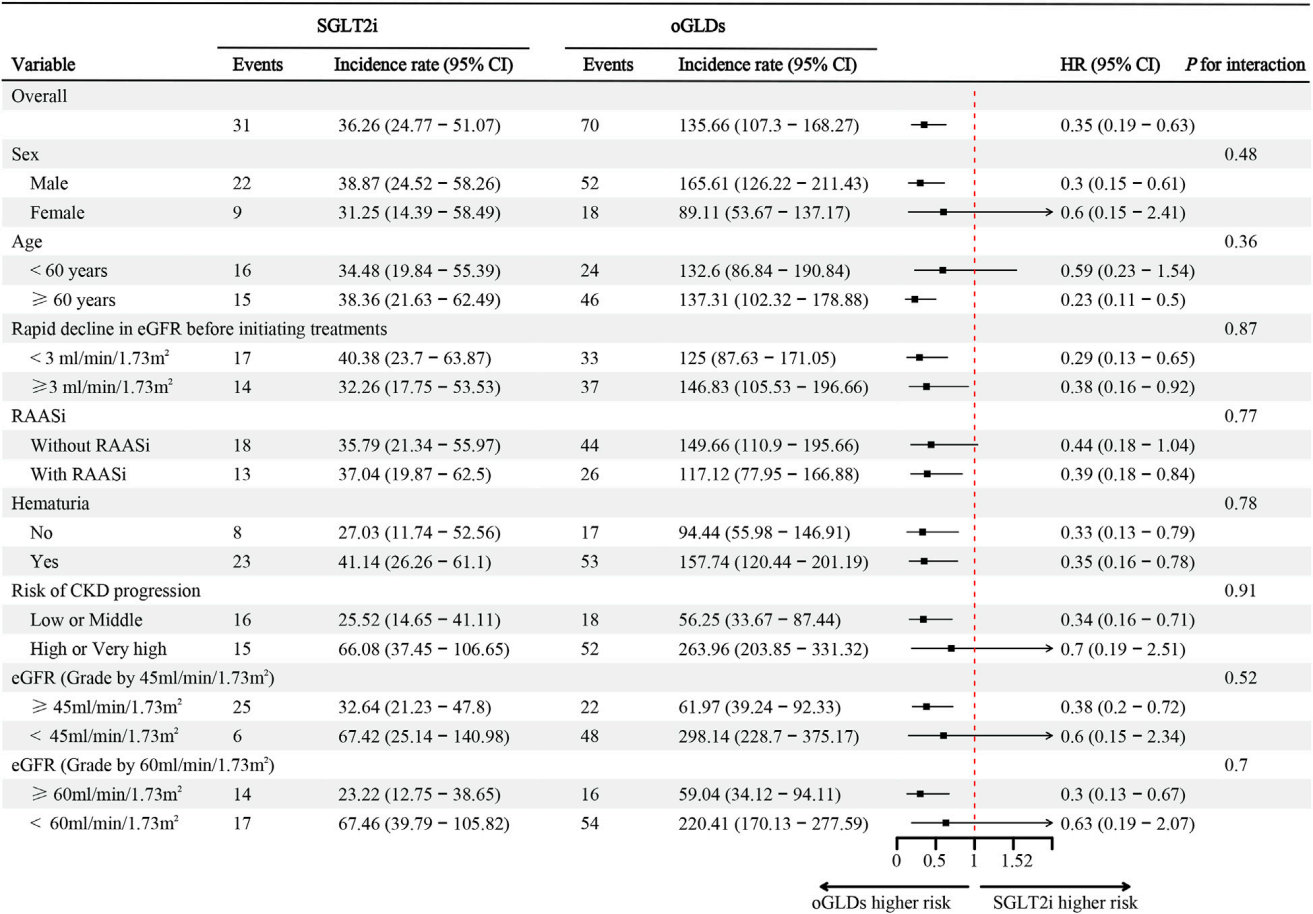
The incidence, corresponding rates, and hazard ratio (ITT analysis) of composite kidney endpoints following initiation of SGLT2i and oGLDs. Incidence rates are expressed per 1,000 person-years.

During the follow-up period, the SGLT2i group experienced 31 cases of kidney composite endpoint events, while the oGLDs group had 70 cases. The cumulative incidence of kidney composite endpoint events was higher in the oGLDs group compared to the SGLT2i group (Figure 3A). The incidence rate of composite endpoint events in the oGLDs group (136/1,000 person-years) was higher than that in the SGLT2i group (36/1,000 person-years) (Figure 4). Regardless of the risk for CKD progression, the cumulative incidence of kidney composite endpoint events remained higher in the oGLDs group compared to the SGLT2i group (Supplementary Figure S6). Patients initiating SGLT2i had a 65% lower risk of kidney composite endpoint events compared to other glucose-lowering drugs (HR 0.35, 95% CI 0.19–0.63) (Figure 4). Subgroup analyses showed no interaction between the use of SGLT2 inhibitors and sex (“female” vs “male”), age (“<60” vs “≥60” years), eGFR (“<60” vs “≥60” mL/min/1.73 m^2^), eGFR (“<45” vs “≥ 45”mL/min/1.73 m^2^), hematuria (“yes” vs “no”), pre-treatment with RAASi (“yes” vs “no”), rapid eGFR decline (“yes” vs “no”), and prognostic grade of CKD (“low or middle” vs “high or very high”) (all *p* > 0.36) (Figure 4).

During the follow-up, 1 case of ESRD occurred in the SGLT2i group, with 30 patients experiencing a decline in eGFR exceeding 30%. In contrast, in the oGLDs group, 46 cases of ESRD occurred, with 24 patients experiencing a decline in eGFR exceeding 30% (Supplementary Figures S7, S8). The cumulative incidence of eGFR decline exceeding 30% and ESRD was consistently higher in the oGLDs group compared to the SGLT2i group (Figure Figures 3B, C). Initiation of SGLT2i was associated with a lower risk of a decline in eGFR exceeding 30% (HR 0.37, 95% CI 0.20–0.68) and ESRD (HR 0.11, 95% CI 0.02–0.68) compared to oGLDs (Supplementary Figures S7, S8).

## 4 Discussion

This study demonstrate that initiation of SGLT2i significantly delays the decline of eGFR in mainland China, as compared to oGLDs. Furthermore, patients using SGLT2i exhibit a substantial reduction in the risk of major kidney events when compared to those using oGLDs. It is noteworthy that these results remain consistent across patients with varying risks of CKD progression. Although the efficacy of SGLT2i in slowing down the decline of eGFR is evident across different populations, there exists heterogeneity in its effects due to variations in subgroups.

The exact mechanisms by which SGLT2 inhibitors protect the kidneys are not yet fully understood. One primary mechanism proposed involves the enhancement of tubuloglomerular feedback, which occurs when increased sodium reaches the distal tubule, particularly the macula densa. This results in the constriction of afferent arterioles, thereby reducing glomerular hyperfiltration. Another closely related mechanism is the suppression of the renal renin–angiotensin–aldosterone system, which further decreases glomerular hyperfiltration and intraglomerular pressure. Additional mechanisms that have been highlighted in various research studies include (i) a slight rise in the production of ketone bodies like β-hydroxybutyrate, which can serve as alternative energy sources for ATP synthesis in mitochondria, thus reducing inflammation, and (ii) a reduction in hypoxia, oxidative stress, and fibrosis (Belančić and Klobučar, 2023). Numerous RCTs have confirmed the kidney protective effects of SGLT2i, including reducing proteinuria, slowing the decline of eGFR, and lowering the risk of ESRD (Perkovic et al., 2019; Herrington et al., 2023; Heerspink et al., 2020a; Kaze et al., 2022). However, considering the strict inclusion criteria of RCTs, the generalizability of their conclusions is limited, thus emphasizing the increasing recognition of the importance of supplementing RCT findings with real-world data. RWS can explore the effectiveness and safety of medications in real-world settings and apply clinical trial results to diverse patients, aligning more closely with clinical practice scenarios (Sherman et al., 2016). To our knowledge, there have been several real-world investigations conducted in the regions of Hong Kong and Taiwan in China, exploring the impact of SGLT2i on kidney outcomes in T2D and CKD (Au et al., 2022; Chang et al., 2022; Wong et al., 2021; Tang et al., 2021; Lui et al., 2022). However, there are few studies on the influence of SGLT2i on kidney outcome in patients with T2D and CKD in mainland China. Additionally, variations in healthcare standards across different regions, patient compliance, extent of medical insurance coverage, and genetic disparities among ethnicities may contribute to divergent therapeutic outcomes of SGLT2i in the real world. Therefore, for the above factors, we conducted this study.

Limited research has been conducted on the alterations in the slope of eGFR decline following the use of SGLT2i in RWS. Nagasu et al. (2021) revealed a difference in the rate of decline in eGFR between the group initiate with SGLT2 inhibitors and the group treated with oGLDs, with a value of 0.75 mL/min/1.73 m^2^ per year (95% CI, 0.51–1.00) in patients with T2D and CKD. In the CVD-REAL 3 study (Heerspink et al., 2020b), which included patients with T2D, the final results showed a difference in the rate of eGFR decline between the groups initiated with SGLT2i and oGLDs, with a value of 1.53 mL/min/1.73 m^2^ per year (95% CI, 1.34–1.72). Another real-world study, focusing on patients with T2D, demonstrated a difference in the rate of eGFR decline between the group treated with SGLT2i and the group treated with oGLDs, with a value of 0.76 mL/min/1.73 m^2^/year (95% CI, 0.24–1.28) (Lui et al., 2022). Our study found that the between-group difference in eGFR decline rate was 4.94 mL/min/1.73 m^2^ per year (4.73–5.15), favoring the SGLT2i group.

Compared to other studies (Nagasu et al., 2021; Heerspink et al., 2020b; Lui et al., 2022), our study observed a faster decline in eGFR prior to initial treatment, and the difference in eGFR decline slope between the SGLT2i group and the oGLDs group was also greater after treatment. The CVD-REAL 3 study (Heerspink et al., 2020b) and the study by Lui et al. (2022) included patients with T2D, whose kidney damage was milder, resulting in a slower natural decline in eGFR slope and smaller differences in eGFR decline rate between treatment groups. Similar to the study by Nagasu et al. (2021), we all included patients with T2D and CKD. The slope of eGFR decline in the oGLDs group in our study was much higher as compared with that in the oGLDs group in the study by Nagasu et al. (2021), while among patients initiating SGLT2i was found to be comparable. The slight variations in age, sex, and initial average eGFR levels between the patients enrolled in our study. However, the rate of eGFR decline in patients in the oGLDs group varied considerably between the two studies. This may be due to a higher proportion of patients with eGFR <45 mL/min/1.73 m^2^ and a greater decline in eGFR slope prior to initiation of treatment in our study. Despite the fact that the rate of eGFR decline prior to the initiation of treatment was faster in both groups in our study, and continued to be rapid after the initiation of receiving oGLDs, the rate of eGFR decline in the patients prescribed SGLT2i was significantly delayed, and the difference in the slope of eGFR decline between the two groups was also more pronounced than in previous studies. It implies that SGLT2i exhibit greater efficacy in mitigating the deterioration of eGFR among the populace of mainland China. Nagasu et al. (2021) investigated the impact of initial use of SGLT2i and other oGLDs on the rate of eGFR decline over time in patients, stratified by the presence of proteinuria and rapid eGFR decline. The results demonstrated that regardless of the presence of proteinuria or rapid eGFR decline prior to initiation of treatment, patients in the SGLT2i group exhibited a slower rate of eGFR decline over time. To comprehensively assess the impact of proteinuria and eGFR on the rate of eGFR decline, we classified patients into CKD progression risk categories based on the KDIGO guidelines (KDIGO, 2012). Our findings revealed that irrespective of the CKD progression risk category, patients in the SGLT2i group exhibited a slower rate of eGFR decline over time.

Our study findings indicate that there was no significant decline in eGFR within the initial month of SGLT2i initiation, which contrasts with previous research. This discrepancy may be attributed to several factors. Firstly, the utilization of SGLT2i in China commenced relatively late, with prescription rates only starting in 2019. Given our emphasis on monitoring patient hydration status following SGLT2i use, we routinely advise patients to increase their fluid intake. Secondly, in our study, a considerable number of patients underwent eGFR reevaluation within one to 2 weeks of SGLT2i initiation. In cases where eGFR decline was observed, many patients requested discontinuation of the drug, and in most instances, physicians were unable to alter their decisions. Consequently, these patients, who experienced eGFR decline after discontinuation, were not included in the on-time treatment analysis, thereby minimizing the occurrence of rapid decline within the first month. Therefore, considering the established fact that rapid eGFR decline can occur within the initial month of SGLT2i use, it is unnecessary to conduct frequent follow-up assessments, as this would alleviate significant psychological stress for both patients and physicians. This approach is particularly feasible for patients with stable conditions.

In the subgroup analysis of declining eGFR, the CVD-REAL 3 (Heerspink et al., 2020b) study found consistent results in the differences of eGFR decline between patients initiating SGLT2i and oGLDs, across different HbA1c (“≤53” vs “>53”), eGFR (“<60” vs “60–90” vs “>90” mL/min/1.73 m^2^), presence of cardiovascular disease (“yes” vs “no”), and use of RAAi (“yes” vs “no”). However, Nagasu et al. revealed some heterogeneity, showing consistent deceleration of eGFR decline with SGLT2i use in patients of different ages (“<65” vs “≥65” years), eGFR levels (“<60” vs “≥60” mL/min/1.73 m^2^), and proteinuria status (“yes” vs “no”). Nonetheless, differences were observed in patients with different rates of eGFR decline before the index time (“rapid decline” vs “without rapid decline”) and in those using RAASi (“yes” vs “no”). Notably, SGLT2i demonstrated a more pronounced effect in slowing down eGFR decline in patients without rapid decline and in those using RAASi prior to initiation of index drugs. Our study showed an interaction between the utilization of SGLT2i and variables such as sex, age, rapid decline in eGFR, hematuria, and risk for CKD progression. However, no significant interaction was observed between the use of RASSi (categorized as “yes” vs “no”) or eGFR levels (categorized as “<60” vs “≥60”mL/min/1.73 m^2^) prior to initiation of treatment. Notably, SGLT2i demonstrated better effect in male patients, those under the age of 60, those without rapid eGFR decline, those with hematuria, and those classified as high or very high risk for CKD progression. However, it is important to emphasize that this merely demonstrates the potential superiority of certain subgroups of SGLT2i, whereas in reality, all subgroups of SGLT2i have exhibited a retarding effect on the decline of eGFR.

Indeed, with the exception of the CREDENCE study (Perkovic et al., 2019), the patients enrolled in most RCTs exhibited relatively better renal function compared to RWS. Throughout the observation period, only a few clinically significant endpoints, such as ESRD or a 50% decline in eGFR, occurred. Consequently, the primary outcome measures of these studies mainly focused on changes in eGFR slope. RWS serve to compensate for the lack of significant endpoint events in SGLT2i research findings (Perkovic et al., 2019; Wanner et al., 2016; Perkovic et al., 2018; Mosenzon et al., 2019). Current evidence suggests that the utilization of SGLT2i is associated with a significant reduction in the risk of major kidney endpoints. However, there are also some studies that demonstrate different results. In the multi-center real-world study CVD-REAL 3, the risk reduction for composite kidney endpoints (50% decline in eGFR, ESRD) after SGLT2i use did not exhibit heterogeneity among patients from different countries (Heerspink et al., 2020b).

A recent RWS from Sweden showed that compared to GLP-1RA initiators, there was no significant change in the composite kidney endpoint among SGLT2i initiators (Lugner et al., 2021). Additionally, several studies conducted in Asian populations, including those from Hong Kong and Japan, also suggested that the composite kidney endpoint did not significantly differ among patients using SGLT2i (Wong et al., 2021; Lui et al., 2022; Takeuchi et al., 2020). Conversely, another subset of studies conducted in Asian populations demonstrated a decreased risk of composite kidney endpoint events among patients using SGLT2i (Chang et al., 2022; Koh et al., 2021; Lin et al., 2021; Lin et al., 2019). Although the variations in these results may be attributed to differences in patient characteristics, definitions of composite kidney endpoints, and clinical settings, they also indicate the heterogeneity of treatment effects that may exist among different patient populations following the use of SGLT2i. These studies highlight the inconsistency of results observed in Asian populations, yet there is currently a lack of research investigating the changes in the risk of major kidney endpoint events among Chinese mainland patients with T2D and CKD following the use of SGLT2i. Our study serves as a valuable addition to the literature, demonstrating that for individuals in mainland China, SGLT2i significantly reduces the risk of composite kidney endpoints, ESRD, and a 30% decline in eGFR.

Some studies have demonstrated heterogeneity in the reduction of major kidney endpoints after the use of SGLT2i in subgroup analyses. Idris et al. (2022) found an interaction between the use of SGLT2i and sex in the risk reduction of eGFR decline exceeding 40%. Similarly, Koh et al. (2021) revealed heterogeneity in the interaction between the use of SGLT2 inhibitors and eGFR and body mass index in reducing the risk of ESRD. Au et al. (2022) discovered an interaction between the use of SGLT2 inhibitors and eGFR rapid decline (defined as >4% decline in eGFR per year) in reducing the risk of ESRD. Additionally, Pasternak et al. (2020) found an interaction between the use of SGLT2 inhibitors and major cardiovascular disease in reducing the risk of serious kidney events (renal replacement therapy, death from renal causes, and hospital admission for renal events). However, our study consistently showed the effect of SGLT2 inhibitors in reducing the risk of composite kidney endpoints across all subgroups.

Certainly, this study has several limitations. Firstly, although this is the first RWS conducted in mainland China, it primarily focuses on individuals with T2D and CKD in mainland China. Therefore, caution should be exercised in generalizing these findings to T2D and CKD patients in all regions of China. Secondly, the study did not explore the issue of SGLT2i dosage. However, based on existing research, even with reduced dosage, the benefits of using SGLT2i remain significant. Thirdly, the sample size of this study is relatively small. However, due to the rapid prescription of SGLT2i in T2D patients, the number of patients not using SGLT2i is even smaller, resulting in a limited number of patients in oGLDs groups. Fourthly, we cannot completely rule out the interference of unknown confounding factors in the design of this retrospective study. Fifthly, adverse event information related to the use of SGLT2i was not collected in this cohort. Sixth, the results of this study may only be applicable to DKD patients in less economically developed areas of China, such as southwest China. In addition, Since SGLT2i were introduced into China at a later time, the follow-up period for the SGLT2i group is relatively shorter than that for oGLDs group. Lastly, due to missing ACR data, interpolating ACR values may compromise the integrity of the data and the inverse probability weighting score. Therefore, we employed protein examination in urine routine as a measure of the severity of proteinuria in patients.

In summary, our study presents real-world data on kidney outcomes in a population with T2D and CKD in mainland China after the use of SGLT2i. Patients treated with SGLT2i exhibited a slower decline in eGFR compared to those treated with oGLDs, and also experienced a reduced risk of composite kidney endpoint events.

## Data Availability

The original contributions presented in the study are included in the article/Supplementary Material, further inquiries can be directed to the corresponding author.

## Abbreviations

SGLT2i, Sodium-dependent glucose cotransporters inhibitors; T2D, Type 2 Diabetes; CKD, Chronic Fidney Disease; oGLDs, other Glucose-Lowering Drugs; ESRD, End-Stage Renal Disease; RWS, Real World Studies; RCTs, Randomized Controlled Trials; ACR, Albumin-to-Creatinine Ratio; eGFR, estimated Glomerular Filtration Rate; IPTW, Inverse Probability of Treatment Weighting (IPTW); SMD, Standardized Mean Difference (SMD); RAASi, Renin-angiotensin-aldosterone system inhibitors (RAASi); LDL-c, LDL-cholesterol; ACEi, Angiotension Converting Enzyme inhibitors; ARB, Angiotensin Receptor Blocker.
