# Supplementary figures and images for: Kidney outcomes associated with SGLT2 inhibitors compared to other glucose-lowering drugs: a real-world study from China
Source: Front Pharmacol. 2024 Dec 3;15:1468435. doi: 10.3389/fphar.2024.1468435 (PMC11649429; doi:10.3389/fphar.2024.1468435)

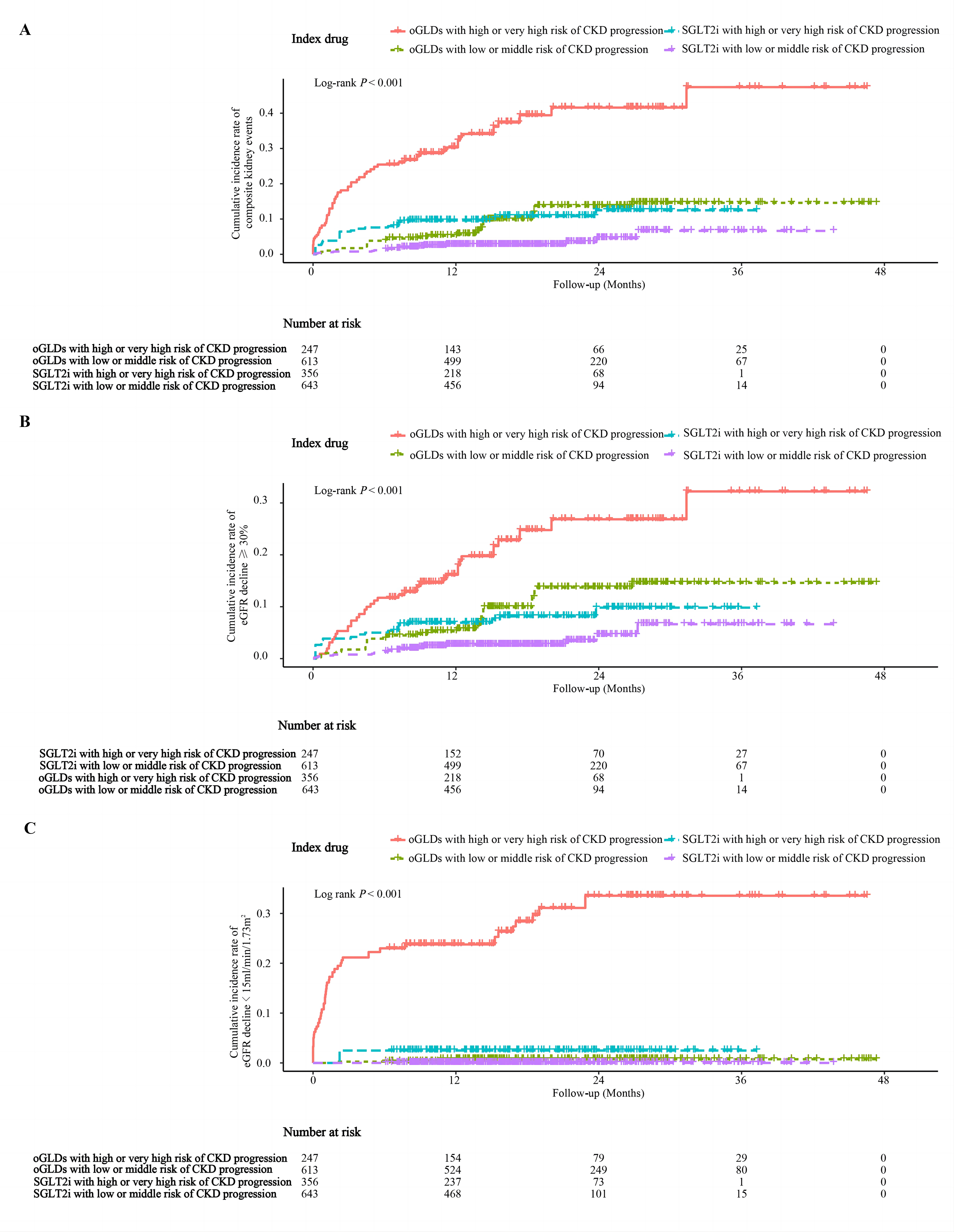

Supplement: Supplementary file 1 [file Image6.TIF]

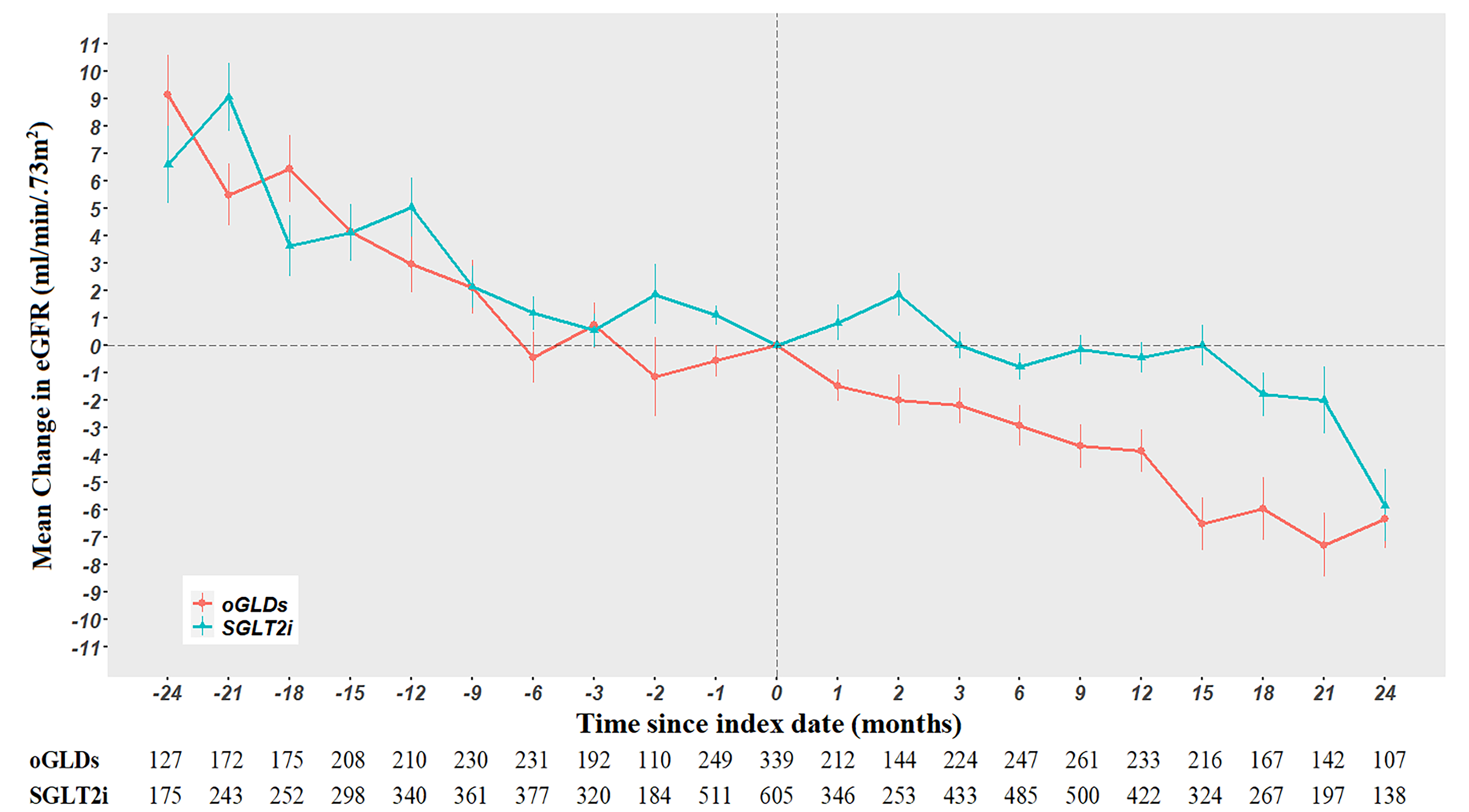

Supplement: Supplementary file 2 [file Image3.TIF]

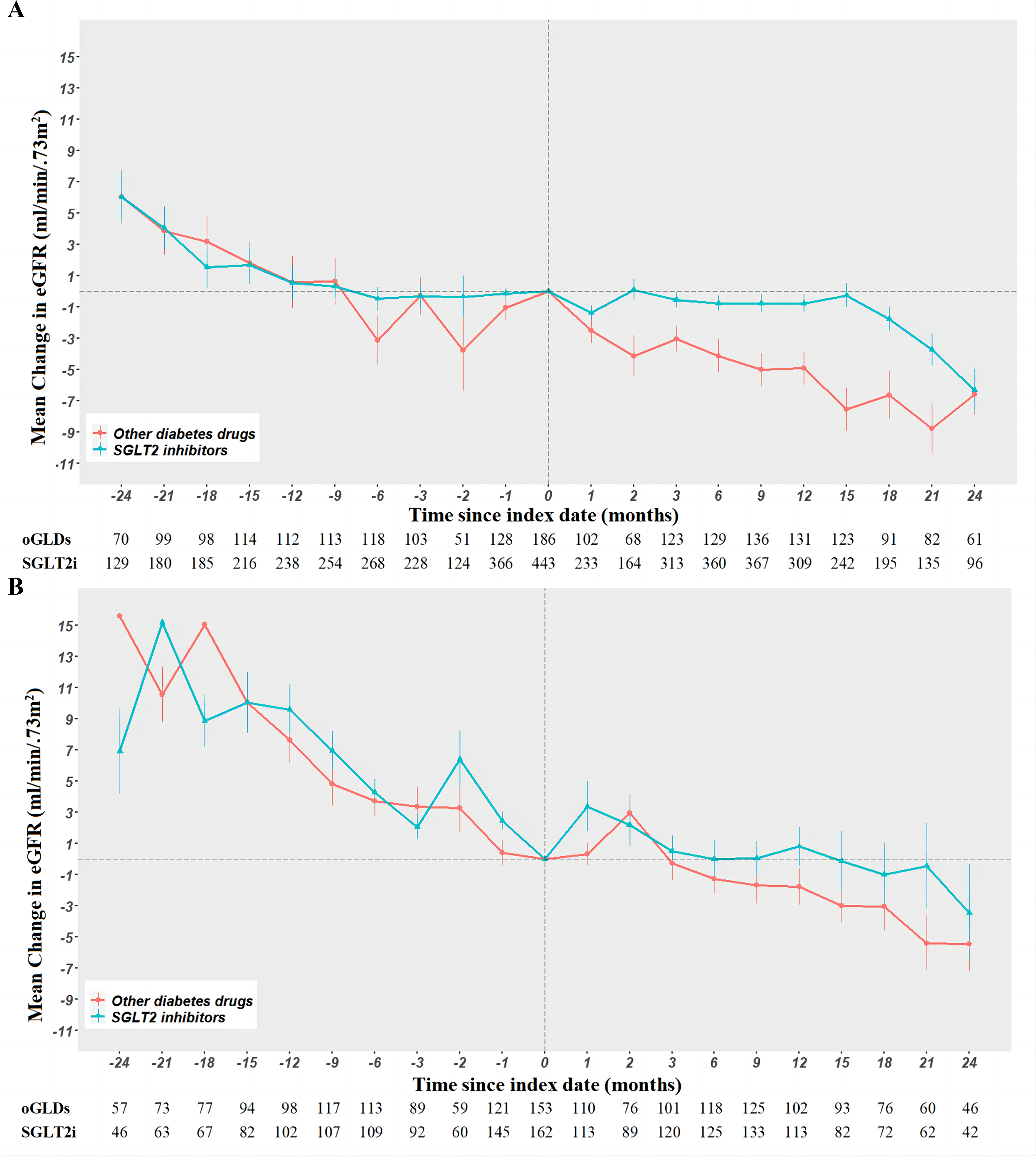

Supplement: Supplementary file 3 [file Image4.TIF]

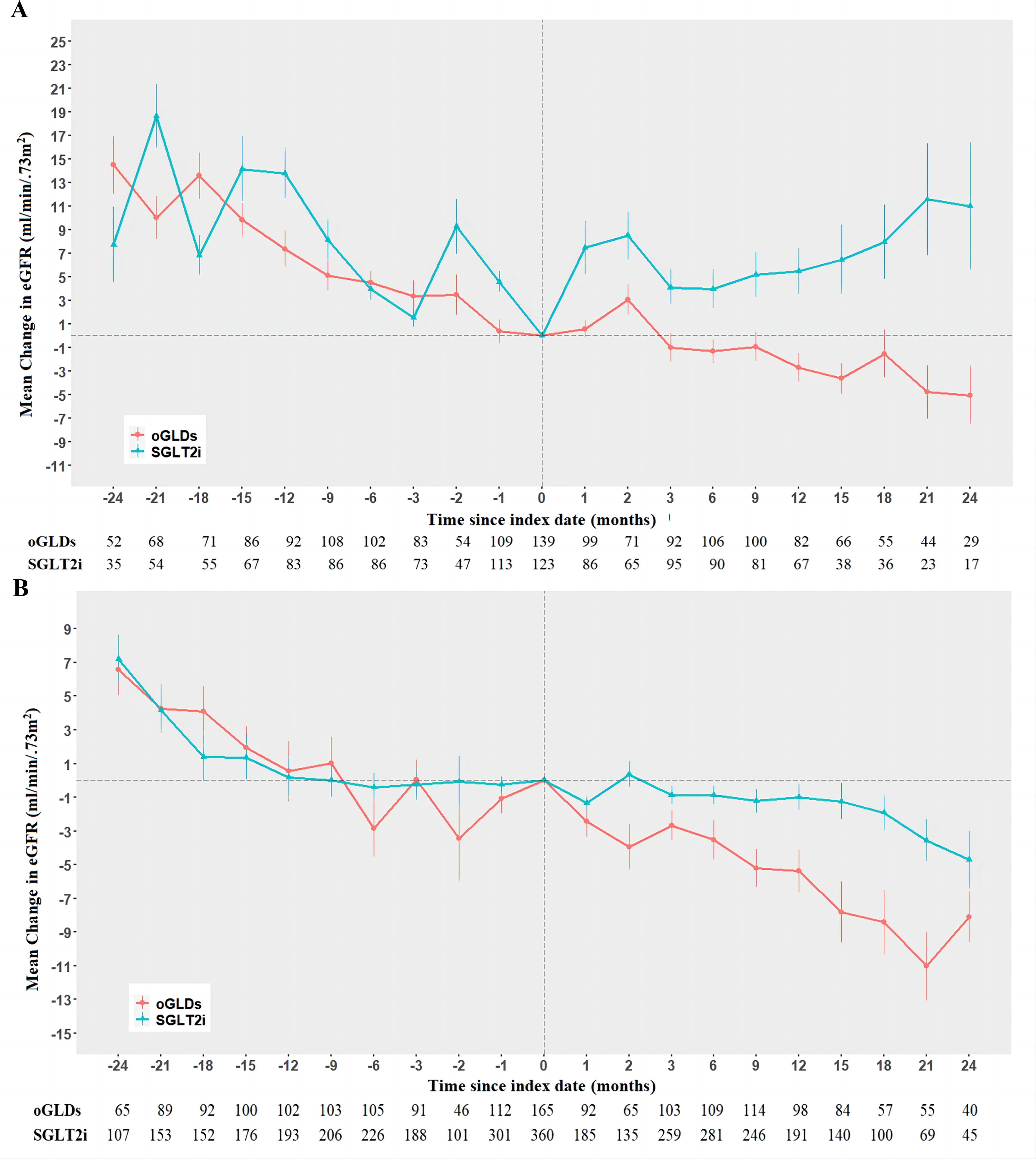

Supplement: Supplementary file 4 [file Image2.TIF]

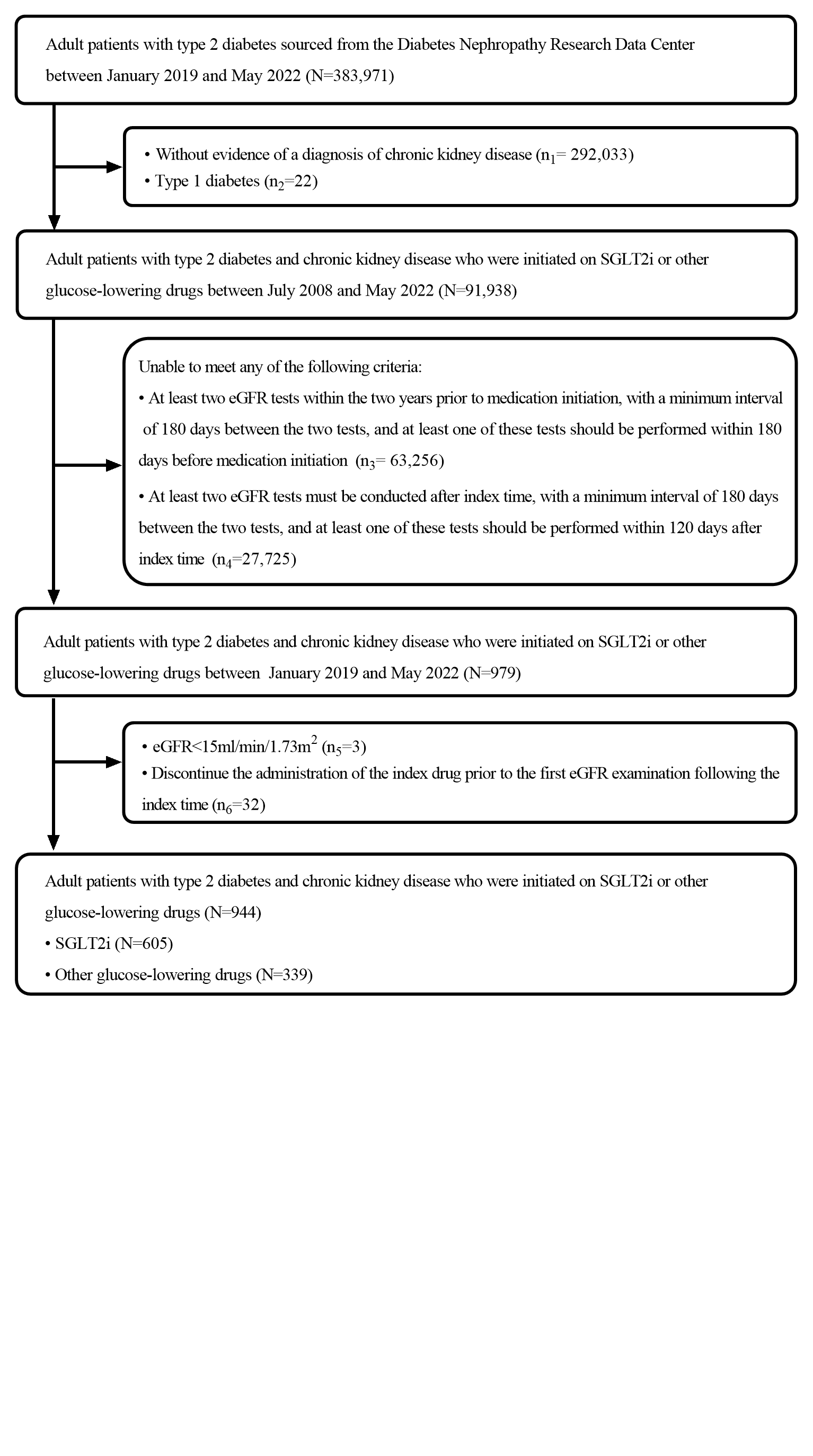

Supplement: Supplementary file 5 [file Image1.TIF]

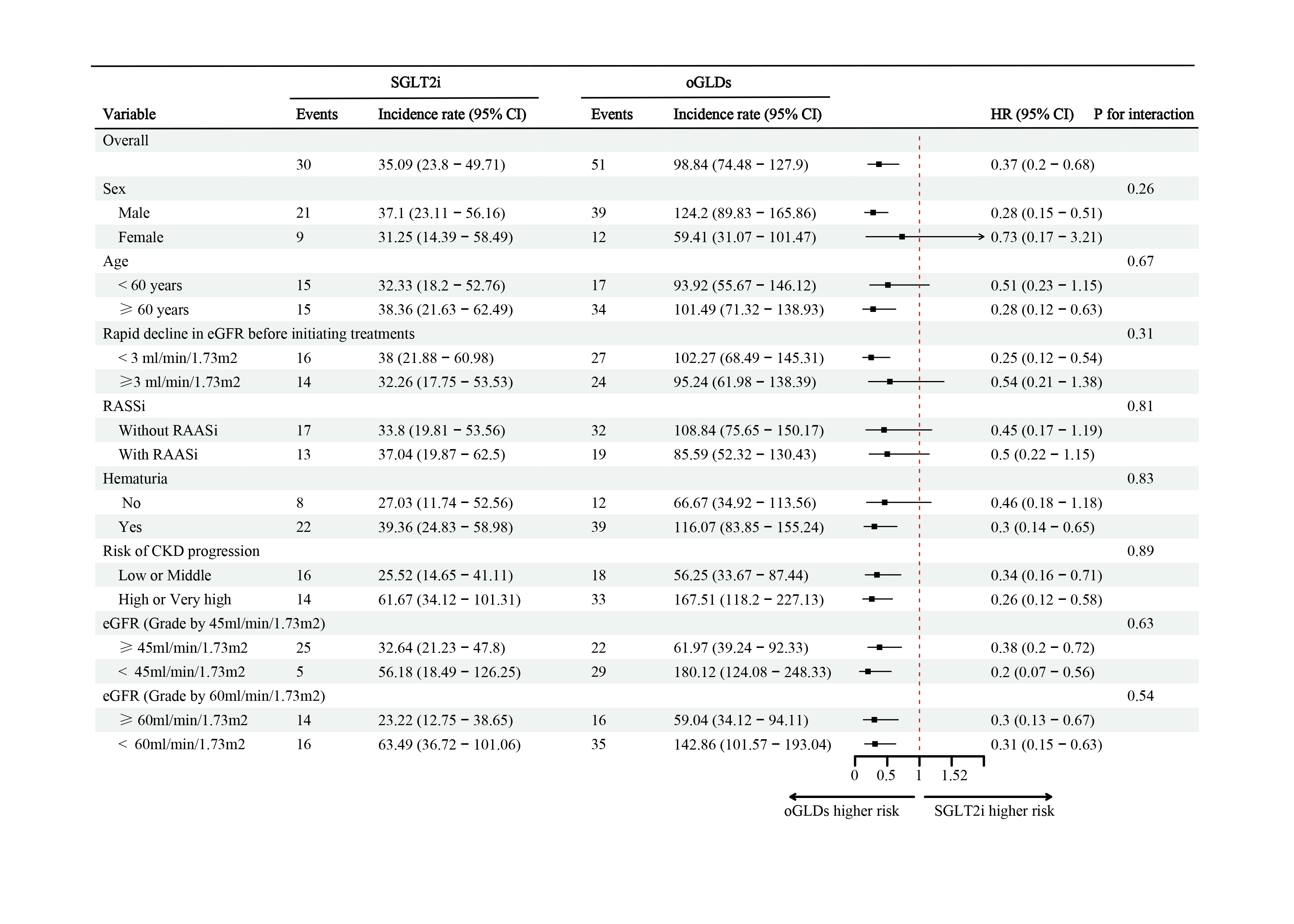

Supplement: Supplementary file 6 [file Image7.TIF]

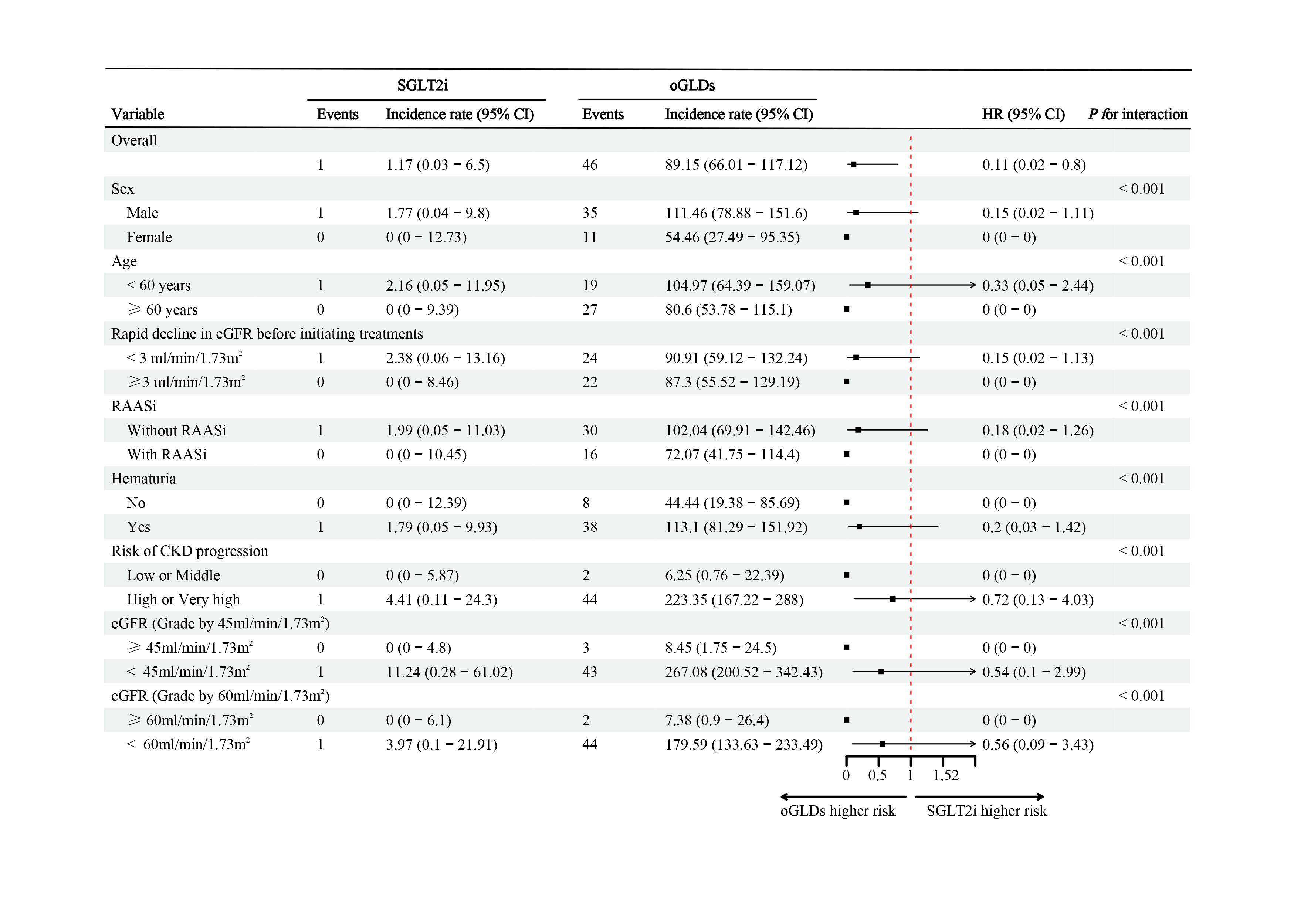

Supplement: Supplementary file 8 [file Image8.TIF]

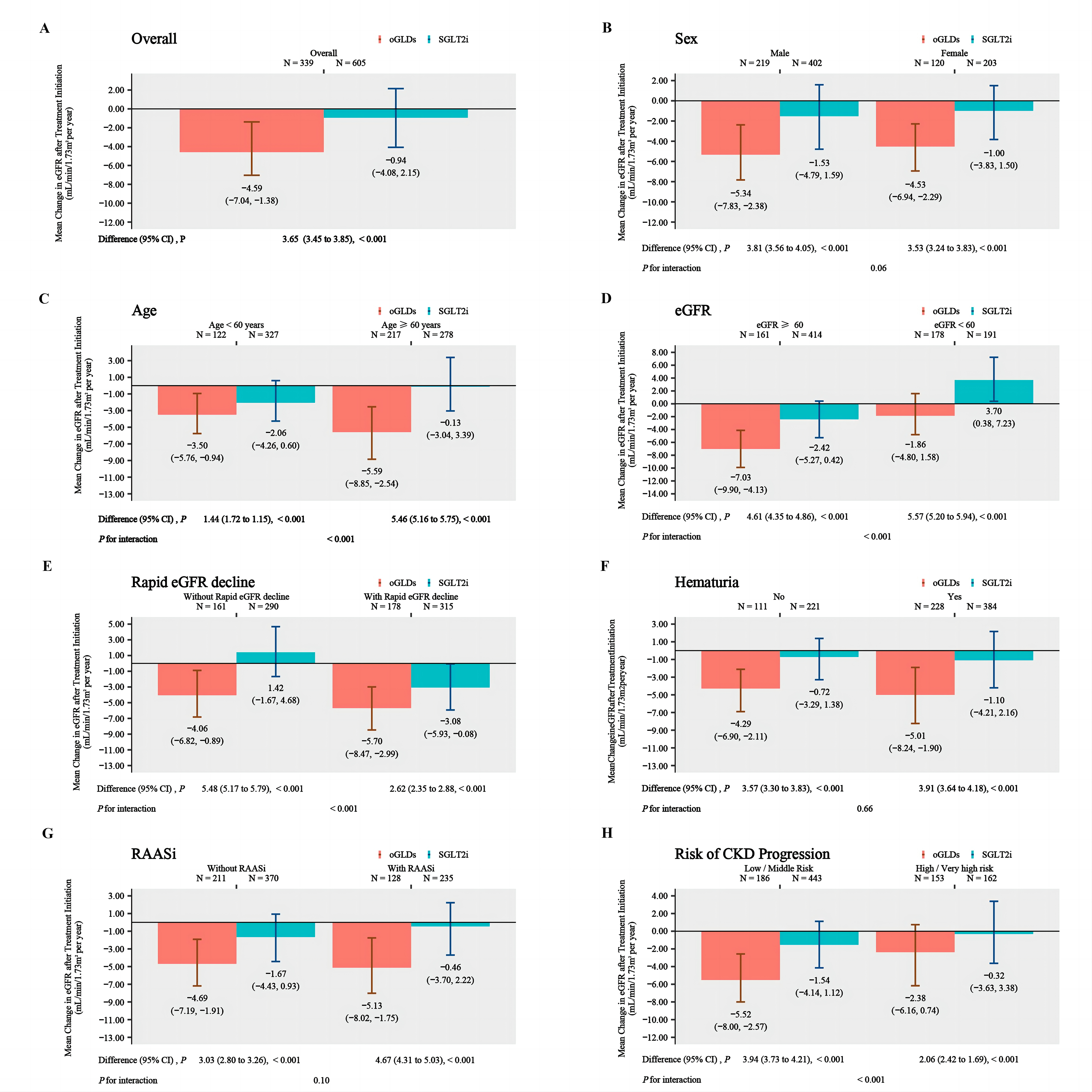

Supplement: Supplementary file 9 [file Image5.TIF]
