# Supplementary material for: Kidney outcomes associated with SGLT2 inhibitors compared to other glucose-lowering drugs: a real-world study from China
Source: Front Pharmacol. 2024 Dec 3;15:1468435. doi: 10.3389/fphar.2024.1468435 (PMC11649429; doi:10.3389/fphar.2024.1468435)
Supplement: Supplementary file 7 [file DataSheet1.pdf]

Supplementary Material

Inclusion criteria: 1) Age > 18 years, 2) Diagnosis of T2DM and / or prescribed glucose-lowering drugs, 3) Meets the diagnostic criteria for CKD: ① Routine urine examination.: protein ≥ 1+ (dipstick test), ② Urine albumin-to-creatinine ratio (ACR) ≥ 30mg/g, ③ Urine total protein-to-creatinine ratio (TPCR) ≥ 0.045g/mmol, ④ 24-hour urine total protein (24h-TP) ≥ 0.15g/24 hours, ⑤ eGFR < 60ml/min/1.73m^2, meet one of the above and with abnormal values observed on two or more times.

Exclusion criteria: 1) Type 1 diabetes, 2) Unable to meet any of the following criteria: ① At least two eGFR tests within the two years prior to medication initiation, with a minimum interval of 180 days between the two tests, and at least one of these tests should be performed within 180 days before medication initiation, ② At least two eGFR tests must be conducted after index time, with a minimum interval of 180 days between the two tests, and at least one of these tests should be performed within 120 days after index time, 3) eGFR<15ml/min/1.73m^2, 4) Discontinue the administration of the index drug prior to the first eGFR examination following the index time.

Supplementary Table 1. Clinical characteristics at index date before IPTW (Propensity Score)

Characteristics	All (n=951)	SGLT2i group (n=605)	oGLDs group (n=339)	SMD (%)
Age, mean (SD), years	61.84 (13.47)	59.93 (13.25)	65.24 (13.22)	40.1
Sex, Male, [n (%)]	621 (65.8)	402 (66.4)	219 (64.6)	3.9
Hemoglobin A1c, mean(SD), %	7.16 (1.35)	7.19 (1.36)	7.12 (1.33)	5.5
Serum albumin, mean(SD), g/L	43.97 (5.07)	44.48 (4.74)	43.06 (5.50)	27.6
Hemoglobin, mean (SD), g/L	132.95 (22.16)	137.34 (20.89)	125.14 (22.23)	56.6
Triglyceride, mean (SD), mmol/L	2.13 (2.20)	2.26 (2.50)	1.89 (1.46)	8.5
Total cholesterol, mean (SD), mmol/L	4.46 (1.32)	4.50 (1.23)	4.39 (1.46)	18.3
LDL-c, mean (SD), mmol/L	2.49 (2.20)	2.53 (0.98)	2.43 (1.13)	9.9
ACEi, [n (%)]	57 (6.0)	35 (5.8)	22 (6.5)	2.9
ARB, [n (%)]	320 (33.9)	207 (34.2)	113 (33.3)	1.9
Statin, [n (%)]	334 (35.4)	213 (35.2)	121 (35.7)	1
Proteinuria classification [n (%)]	603 (63.4)	393 (64.4)	208 (61.0)	8.9
— or ±	488 (51.7)	317 (52.4)	171 (50.4)	
1+ or 2+	348 (36.9)	228 (37.7)	120 (35.4)	
3+ or 4+	108 (11.4)	60 (9.9)	48 (14.2)	
Hematuria, [n (%)]	612 (64.8)	384 (63.5)	228 (67.3)	9.9
Glucose-lowering drugs, [n (%)]				
Metformin	397 (42.1)	255 (42.1)	142 (41.9)	0.5
DPP4i	330 (35.0)	169 (27.9)	161 (47.5)	41.2
Sulfonylurea	253 (26.8)	137 (22.6)	116 (34.2)	25.8
GLP-1RA	21 (2.2)	18 (3.0)	3 (0.9)	15.2
Thiazolidinedione	23 (2.4)	19 (3.1)	4 (1.2)	13.5
AGI	127 (13.5)	48 (7.9)	79 (23.3)	43.3
Insulin	272 (28.8)	144 (23.8)	128 (37.8)	30.6
Hypertension, [n (%)]	432 (45.8)	280 (46.3)	152 (44.8)	2.9
eGFR, mL/min/1.73 m2	68.87 (28.88)	74.86 (24.83)	58.19 (32.33)	58.2
eGFR ≥60 mL/min/1.73 m2, [n (%)]	575 (60.9)	414 (68.4)	161 (47.5)	43.4
eGFR <60 mL/min/1.73 m2, [n (%)]	369 (39.1)	191 (31.6)	178 (52.5)	43.4
eGFR 45–59 mL/min/1.73 m2, [n (%)]	171 (18.1)	123 (20.3)	48 (14.2)	16.4
eGFR <45 mL/min/1.73 m2, [n (%)]	198 (21.0)	68 (11.2)	130 (38.3)	66
Rate of eGFR change prior to index, mean (SD), mL/min/1.73 m2 per year	-3.06 (13.50)	-2.99 (13.35)	-3.19 (13.79)	1.5

IPTW, Inverse Probability of Treatment Weighting; SGLT2i, Sodium-glucose cotransporter-2 inhibitors; SMD, Standardized mean Difference; SD, Standard Deviation; ACEi, Angiotension Converting Enzyme inhibitors; ARB, Angiotensin Receptor Blocker; eGFR, estimated Glomerular Filtration Rate;LDL-c, low density lipoprotein cholesterol; DPP4i, dipeptidyl peptidase-4 inhibitors; GLP-1RA, glucagon-like peptide-1 receptor agonists; AGI, α-glucosidase inhibitor; A standardized difference >10% is considered a nonnegligible difference.

Supplementary Figure 1. Flowchart for inclusion.

Supplementary Figure 2. Temporal eGFR changes among subgroups with different risks of CKD progression before and after the initiation of SGLT2i and oGLDs (On time treatment): low or middle risk (A), high or very high risk (B).

Supplementary Figure 3. Temporal eGFR changes before and after the initiation of SGLT2i and oGLDs (ITT analysis).

Supplementary Figure 4. Temporal eGFR changes among subgroups with different risks of CKD progression before and after the initiation of SGLT2i and oGLDs (ITT analysis): low or middle risk (A), high or very high risk (B).

Supplementary Figure 5. The annual eGFR slope in subgroups following the initiation of SGLT2i and oGLDs (ITT analysis): all (A), sex ('Female' vs. 'Male') (B), age ('< 60' vs. '≥ 60' years) (C), eGFR ('< 60' vs. '≥ 60' ml/min/1.73m^2) (D), rapid eGFR decline ('yes' vs. 'no') (E), RBC ('< 3' vs. '≥3' /HP) (F), RAASi ('yes' vs. 'no') (G), risk of CKD progression ('low or middle' vs. 'high or very high') (H).

Supplementary Figure 6. Cumulative incidence rates of major renal events following initiation of SGLT2i and oGLDs among subgroups with different risks of CKD progression (ITT analysis): The cumulative incidence rates of kdney composite events (A), eGFR decline ≥ 30% (B), and ESRD (C).

Supplementary Figure 7. The incidence, corresponding rates, and hazard ratio (ITT analysis) of eGFR decline ≥ 30% following initiation of SGLT2i and oGLDs. Incidence rates are expressed per 1000 person-years.

Supplementary Figure 8. The incidence, corresponding rates, and hazard ratio (ITT analysis) of ESRD following initiation of SGLT2i and oGLDs. Incidence rates are expressed per 1000 person-years.
